# Post-CD19 Chimeric Antigen Receptor T-Cell Therapy Cytomegalovirus Retinitis

**DOI:** 10.7759/cureus.23002

**Published:** 2022-03-09

**Authors:** Haifa Bin dokhi, Amjad O Alharbi, Nida H Ibnouf, Bader Alahmari, Mohammed N Refka

**Affiliations:** 1 College of Medicine, King Saud Bin Abdulaziz University for Health Sciences, Riyadh, SAU; 2 Collage of Medicine, Alfaisal University, Riyadh, SAU; 3 Oncology, King Abdulaziz Medical City, Riyadh, SAU; 4 Ophthalmology, King Abdulaziz Medical City, Riyadh, SAU

**Keywords:** intravitreal injection, immunodeficiency, approved car-t therapies, retinitis, cytomegalovirus (cmv)

## Abstract

The purpose of this case report was to present a case of cytomegalovirus (CMV) retinitis in a patient with diffuse large B-cell lymphoma (DLBCL) post-CD19 chimeric antigen receptor (CAR) T-cell therapy. A 43-year-old female patient who was complaining of metamorphopsia and sudden blurring in the vision of her left eye was referred to the ophthalmology department. The patient had DLBCL and was started on systemic chemotherapy, which showed no response to therapy and disease progression. Therefore, she was diagnosed with primary refractory DLBCL and treated with CAR T-cell therapy.

The visual acuity of the left eye was 20/25 in the left eye on the Snellen visual acuity chart. The dilated fundus examination of the left eye demonstrated a diffuse yellowish retinal infiltration radiating from the optic disc involving the inferior macula and inferotemporal arcade. A color fundus image of the left eye showed a creamy infiltrate involving the inferior half of the macula sparing the fovea with subtle small white lesions in the midperiphery. Horizontal cross-section optical coherence tomography (OCT) of the macula of the left eye showed islands of destruction of all the retinal layers, which are replaced with moderately hyperreflective material; these infiltrates spare the fovea but with subfoveal fluid. Further systemic evaluation indicated CMV viremia reactivation and an absolute CD4+ cells count of 13 cells/mcL. Thus, she was diagnosed with CMV retinitis.

After three days of the initial presentation, she received the first intravitreal ganciclovir injection; 17 days after presentation, she received five intravitreal ganciclovir injections. The patient responded well to intravitreal ganciclovir therapy. She regained very good vision, and the visual acuity was 20/20 in both eyes. Early recognition and initiation of proper treatment are crucial. Thus, any visual complaints in patients with immunodeficiency should be taken seriously and should be further assessed. As the right eye had retinal scaring indicating previous retinitis, prophylactic treatment with ganciclovir could have been used to reduce the risk of retinitis development in the left eye.

## Introduction

Cytomegalovirus (CMV) is a member of the Herpesviridae family that causes lifelong latent infection within the host after the primary infection [1,2]. Reactivation of CMV can occur in patients with compromised immunity, either iatrogenic or secondary to systemic diseases. This includes patients with primary immunodeficiency disease, acquired immunodeficiency syndrome (AIDS), malignancies, and solid organ transplants or patients following chemotherapy or transplantation of bone marrow. Patients experiencing CMV reactivation may present with generalized signs and symptoms, various site-speciﬁc CMV diseases, or life-threatening disseminated infection [3,4]. CMV retinitis is a serious opportunistic ocular infection that can potentially cause irreversible vision loss [5]. Here, we present a case of CMV retinitis post-CD19 chimeric antigen receptor (CAR) T-cell therapy for diffuse large B-cell lymphoma (DLBCL). It is a form of immunotherapy in which T-cells are collected from the patient and then genetically engineered to produce an artificial receptor. The receptors enable CAR T-cells to target specific antigens on the patient’s diseased or tumor cells. CAR T therapy is approved by the FDA to treat aggressive and refractory non-Hodgkin lymphoma [6]. To our knowledge, CMV retinitis following CAR T-cell therapy has not been reported in the literature yet.

## Case presentation

A 43-year-old female patient was referred from the hematology department complaining of metamorphopsia and sudden blurring in the vision of her left eye. Two years ago, the patient was diagnosed with DLBCL via left cervical lymph node biopsy (stage III AS) and started on systemic chemotherapy. Despite completing six cycles of R-CHOP (rituximab-cyclophosphamide-hydroxydaunorubicin-oncovin-prednisone), two cycles of R-ESHAP (rituximab, etoposide, methylprednisolone, cytarabine, cisplatin), and one cycle of R-ICE (rituximab, ifosfamide, carboplatin, etoposide), there was no response to therapy, and the assessment showed disease progression. Therefore, the patient was diagnosed with primary refractory DLBCL and referred abroad for CD19 CAR T-cell therapy. After completing CAR T-cell therapy on February 25, 2020, she achieved complete remission.

The patient was found to have CMV viremia reactivation with increased CMV quantitative titers (43,054 copies/mL) and an absolute CD4+ cell count of 13 cells/mcL. The cause of CD4+ lymphocytopenia was unexplained. The patient was then admitted and started on IV ganciclovir (5 mg/kg q12 h). After five days of receiving IV ganciclovir, the patient complained of visual symptoms. Based on the initial ophthalmologic examination, the visual acuity was 20/20 in the right eye, and 20/25 in the left eye on the Snellen visual acuity chart. The intraocular pressure (IOP) was 14 mmHg and 15 mmHg in the right and left eyes, respectively. The anterior segment was normal in both eyes.

**Figure 1:**
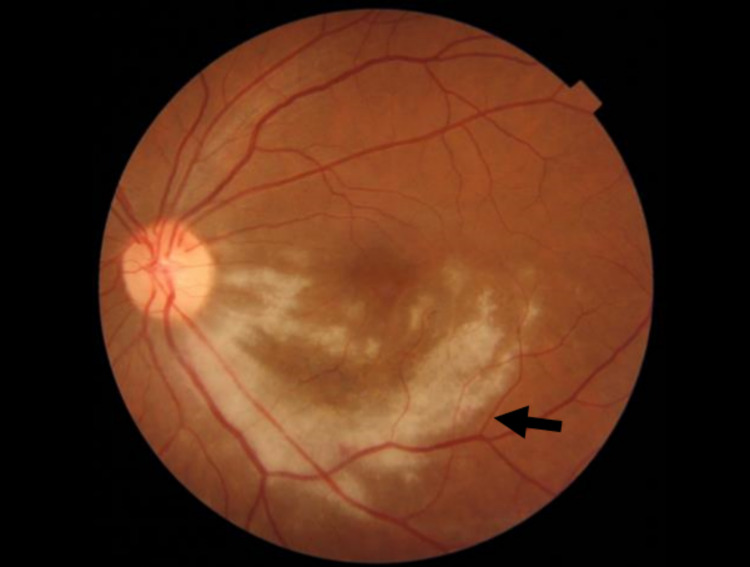
A dilated fundus examination of the left eye

**Figure 2:**
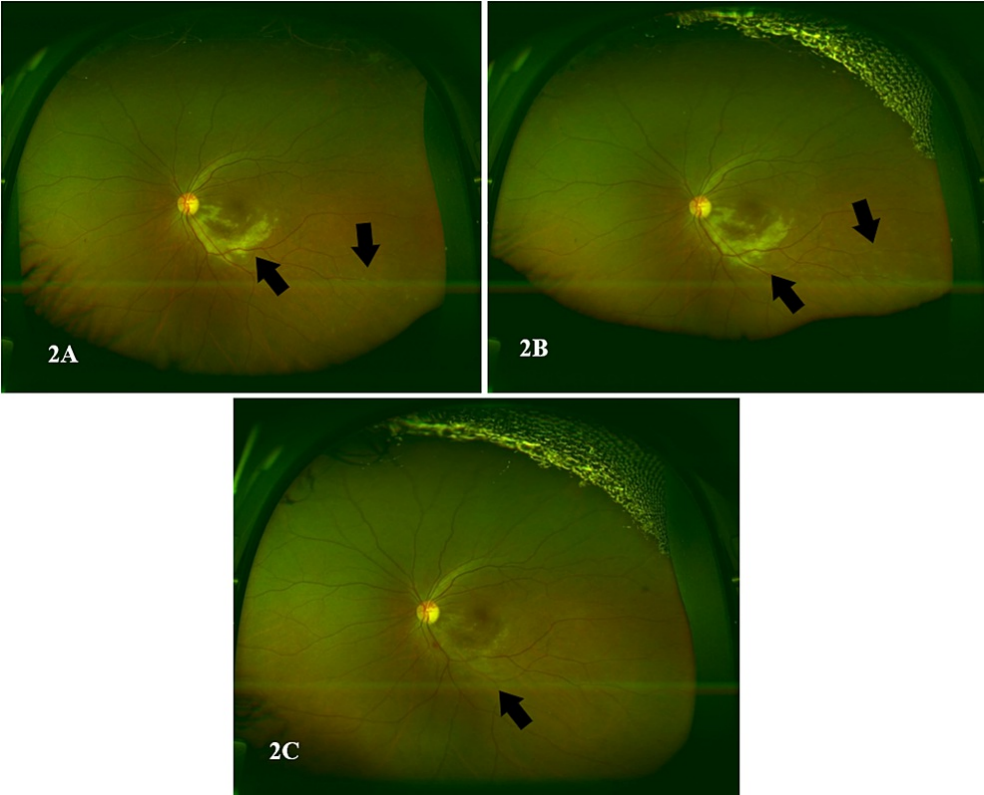
Optos color fundus photo of the left eye (A) Optos color fundus photo of the left eye upon presentation shows white creamy infiltrate involving the inferior half of the macula sparing the fovea with subtle small white lesions in the midperiphery. (B) Three days later (the day of the second intravitreal ganciclovir injection), almost the same central retinitis but the peripheral white spots are more prominent. (C) Color fundus photo of the left eye 17 days after presentation and five ganciclovir intravitreal injections, almost inactive macular lesion and all the peripheral retinitis spots had disappeared.

The dilated fundus examination of the left eye demonstrated a diffuse yellowish retinal infiltration radiating from the optic disc involving the inferior macula and inferotemporal arcade with subfoveal fluid. A color fundus image of the left eye showed a creamy infiltrate involving the inferior half of the macula sparing the fovea with subtle small white lesions in the midperiphery (Figures 1, 2 [Panel A]).

**Figure 3:**
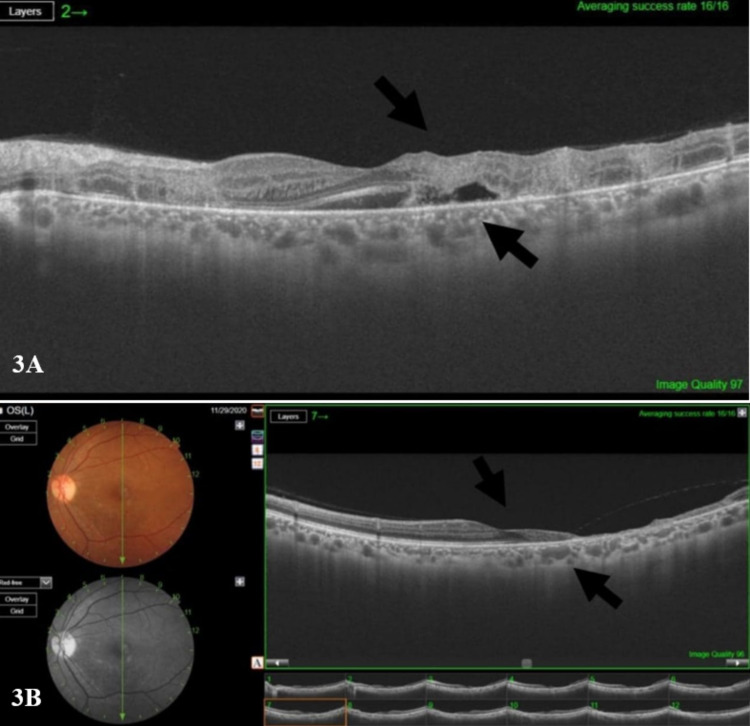
Optical coherence tomography of the macula of the left eye (A) OCT horizontal cross-section of the macula of the left eye. It shows islands of destruction of all the retinal layers, which is replaced with moderately hyperreflective material; these infiltrates spare the fovea but with subfoveal fluid. (B) Vertical OCT cross-section four months post the first injection. It shows almost total loss of retinal layers in the inferior macula with a preserved fovea that explains her good vision. OCT: Optical coherence tomography.

Horizontal cross-section OCT of the macula of the left eye showed islands of destruction of all the retinal layers, which is replaced with moderately hyperreflective material; these infiltrates spare the fovea but with subfoveal fluid (Figure 3, Panel A).

**Figure 4:**
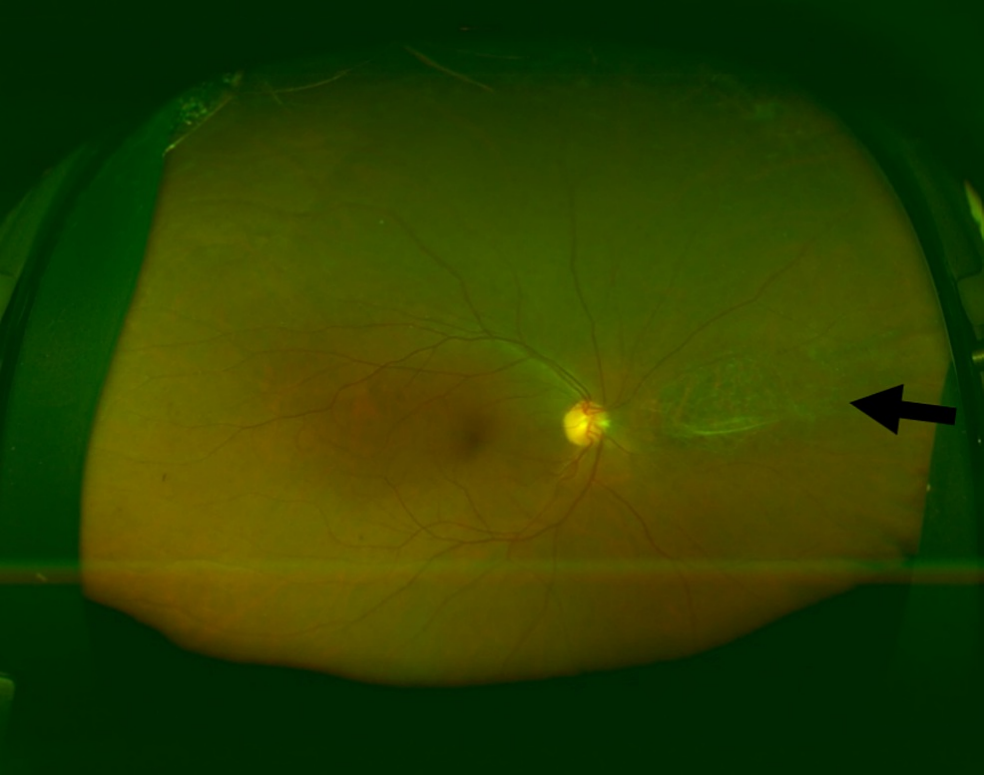
Color fundus photo of the right eye There is a large atrophic scar in the nasal retina, which most likely represents a healed previous CMV retinitis at the presentation. CMV: Cytomegalovirus.

**Figure 5:**
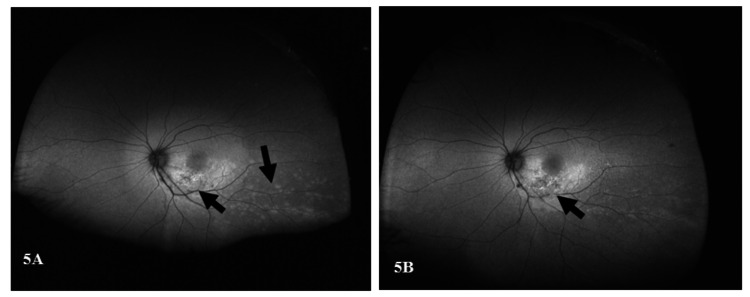
Fundus autofluorescence of the left eye (A) Fundus autofluorescence of the left eye three days after presentation. It shows hyperautofluorescence of the inferior half of the macula corresponding to the active retinitis with a center of hypoautofluorescence (necrotic retina), numerous small hyperfluorescent spots in the midperiphery, and extending toward the periphery in the distribution of the inferotemporal retinal vessels. (B) Fundus autofluorescence photo of the left eye 17 days after presentation. The inferior half of the macula is still hyperautofluorescent, but the central hypoautofluorescent area is bigger. Most of the peripheral lesions are not apparent; some are still slightly hyperautofluorescent with mottling.

A color fundus image of the right eye revealed a large atrophic scar in the nasal retina. The fundus autofluorescence of the right eye showed a large mottled hypo-auto fluorescent lesion nasally, which most likely represents a healed previous CMV retinitis (Figures 4, 5 [Panel A]).

Based on the patient’s clinical presentation and dilated funduscopic examination, she was diagnosed with CMV retinitis. Polymerase chain reaction (PCR) analysis of vitreous and aqueous humor fluids was not performed to confirm CMV retinitis since it was not available during the period of the patient’s presentation. The left eye was then treated with intravitreal ganciclovir injections (2 mg/0.05 ml) twice a week for two weeks and once in the third week while observing the right eye for any signs of retinitis.

Follow-up

Three days after the initial presentation (after receiving the first intravitreal ganciclovir injection), a color fundus photo demonstrated almost the same central retinitis but with more prominent peripheral white spots (Figure 2, Panel B). Moreover, the fundus autofluorescence of the left eye showed hyperautofluorescence of the inferior half of the macula correlating with active retinitis along with a center of hypoautofluorescence suggestive of the necrotic retina. It also revealed numerous small hyperfluorescent spots in the midperiphery that extend toward the periphery in the distribution of the inferotemporal retinal vessels (Figure 5, Panel A).

Seventeen days later (after receiving five intravitreal ganciclovir injections), a color fundus photo showed an almost inactive macular lesion, and all the peripheral retinitis spots had disappeared (Figure 2, Panel C). The inferior half of the macula of the left eye on a fundus autofluorescence is still hyperautofluorescent, but the central hypoautofluorescent area is wider. On the same fundus autofluorescence image, most of the peripheral lesions had disappeared, while some are still slightly hyperautofluorescent with mottling (Figure 5, Panel B).

**Figure 6:**
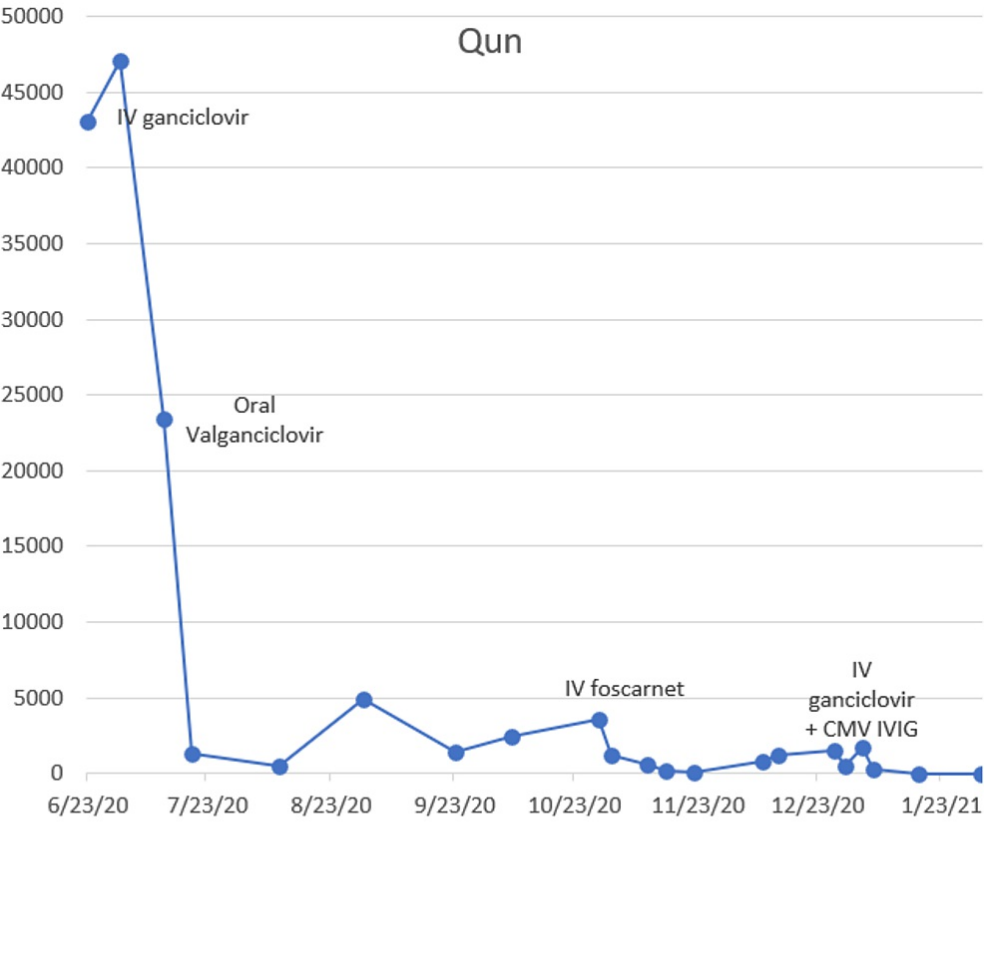
CMV quantitative titers response to antiviral medications CMV: Cytomegalovirus.

After 14 days of initiating ganciclovir, CMV quantitative titers decreased to 23,428 copies/ml. The patient was switched to oral valganciclovir for three months. Although the patient showed a considerable reduction in viral load, she failed to clear the virus totally and continued to show CMV DNA in the blood. CMV quantitative titers were 1580 copies/ml at the end of treatment with valganciclovir. CMV quantitative titers started to increase again (3545 copies/ml), at which point, IV foscarnet was initiated for two months viral load, then dropped significantly to 448 copies/ml for three days, and then increased to 1711 copies/ml. The patient was then scheduled to receive one dose of CMV IVIG in conjugation with eight doses of ganciclovir. Within 20 days of receiving CMV IVIG, the level of CMV DNA in the blood was undetectable (Figure 6).

Outcome

Since receiving CMV IVIG, the level of CMV DNA in the blood was undetectable. The patient responded well to intravitreal ganciclovir therapy. She regained very good vision, and the visual acuity was 20/20 in both eyes. Four months post the first intravitreal injection, vertical cross-section OCT demonstrated an approximately total loss of retinal layers in the inferior macula with the preserved fovea, which explains her good vision (Figure 3, Panel B). The patient is kept on regular follow-up with the continuation of oral valganciclovir and foscarnet. Both eyes are assessed continuously in each visit with dilated fundus examination and OCT.

## Discussion

In immunocompromised patients, CMV can generally exist as a latent intracellular virus that can reactivate if the patient’s immunity is defected. Indeed, this reactivation can cause opportunistic infections of multiple organ systems like the eye, which may present as CMV retinitis [5]. CMV gets to the retina using the hematogenous route and infects the retinal cells through the retinal vascular endothelium [7]. CMV retinitis in its acute phase can manifest as retinal necrosis and secondary inflammatory ischemia, which can result in diffuse intraretinal hemorrhage, retinal whitening and edema, vascular attenuation, and sclerosis. Untreated retinitis can result in retinal necrosis, optic nerve damage, retinal detachment, and blindness [8]. Furthermore, CMV retinitis can be classified into various types. The fulminant type is characterized by edematous hemorrhagic necrosis with white confluent cloudy retinal lesions and the granular type which is usually found in the retinal periphery with less edema and hemorrhage and little to no necrosis. Moreover, perivascular retinitis is characterized by white lesions surrounding the retinal vessels that appear as frosted branches [9,10].

This report represents a case of CMV retinitis post-CD19 CAR T-cell therapy for diffuse large B-cell lymphoma (DLBCL). CAR T-cell therapy is an immunotherapy in which T-cells are collected from the patient and then genetically engineered to enable CAR T-cells to target specific antigens on the patient’s tumor cells [6]. Since CAR T-cell therapy is a novel immunotherapy, its complications, especially eye-related complications, are still not well identified and studied. A recent study investigated the incidence of infections during the first year of CAR T-cell therapy and showed that the cumulative one-year incidence of bacterial and viral infections were 57.2% and 44.7%, respectively. The most common viral pathogen detected was respiratory syncytial virus followed by cytomegalovirus and polyoma BK virus. During the one-year follow-up, cytomegalovirus reactivation (viremia without organ dysfunction) has been reported [11]. This stresses the importance of investigating long-term CAR T-cell therapy outcomes and complications.

The patient regained good vision with a visual acuity of 20/20 after receiving five intravitreal ganciclovir injections. This outcome highlights the importance of early recognition and initiation of proper treatment. Thus, any visual complaints in patients with immunodeficiency should be taken seriously and should be further assessed. In this case, the right eye had retinal scarring indicating the previous retinitis, and prophylactic treatment with ganciclovir could have prevented the retinitis development in the left eye. Further studies are needed to assess CAR T-cell therapy complications. This might help in the initiation of screening protocols that will lead to higher rates of early recognition and proper treatment and thus better outcomes and prognosis.

## Conclusions

This paper describes a case of CMV retinitis post CAR T-cell therapy for diffuse large B-cell lymphoma (DLBCL). A high index of suspicion is needed for any visual symptoms in patients who received CAR T-cell therapy or in patients with immunodeficiency. This necessitates a comprehensive investigation for early detection and treatment. Achieving directed therapy via intravitreal ganciclovir injections along with an interdisciplinary approach resulted in good visual outcomes. Further studies on CAR T-cell therapy complications are crucial for better outcomes.
